# Managing Amitraz Poisoning: Epidemiological Data and Clinical Insights From a Tertiary Care Hospital in Southern India

**DOI:** 10.7759/cureus.72915

**Published:** 2024-11-03

**Authors:** Vishwanath Malkappa Jalawadi, Shravankumar Potkar, Upendra Prasad Yadav, Manjunath S Hiremani

**Affiliations:** 1 Medicine, Shri B. M. Patil Medical College, Hospital and Research Centre, BLDE (Deemed to be University), Vijayapura, IND; 2 Medicine/General Medicine, Shri B. M. Patil Medical College, Hospital and Research Centre, BLDE (Deemed to be University), Vijayapura, IND; 3 Medicine/General Medicine, Narayan Medical College and Hospital, Sasaram, IND; 4 Medical Oncology, Kidwai Memorial Institute of Oncology, Bengaluru, IND

**Keywords:** amitraz, bradycardia, hypertension, hypothermia, metabolic acidosis, miosis, poisoning

## Abstract

Background

Amitraz is a formamidine insecticide and acaricide that acts on α2 adrenergic receptors. There is limited information in the literature regarding the toxicity of this compound and treatment of poisoning. Here, we have studied the epidemiology, symptoms, signs, complications, abnormalities in the tests, management, and prognosis of individuals who were admitted to a tertiary care hospital with acute amitraz intoxication.

Methods

This retrospective study was conducted at a tertiary care facility, Shri B. M. Patil Medical College, Hospital and Research Centre, Vijayapura, India. A total of 76 laboratory-confirmed cases of amitraz poisoning admitted to our hospital between January 2014 and March 2024 were included. All patients were analysed for symptoms, signs, laboratory abnormalities, complications, and management protocols. The data was analysed for frequency, percentage, mean, median, mode, standard deviation, and minimum and maximum values using IBM SPSS Statistics, version 26.

Results

Amitraz poisoning was most commonly observed in patients aged 20-29 years (36.8%); approximately 23.7% were younger than 20 years. The incidence was the lowest (9.2%) in people who were older than 60 years. A total of 97.4% of patients were from rural areas; out of 76, 50% were male patients and the remaining female patients. The most common presentation was vomiting (90.8%), followed by loss of consciousness (31.6%) and drowsiness (23.7%). Miosis was the most common sign seen in 73.7% of patients, 3.9% had mid-dilated pupils, 5.2% had mydriasis, and 17.1% had normal pupils. In total, 32.9% had hypotension, 10.5% had hypertension and 15.8% had hypothermia; 7.9% of patients had pneumonitis as found on chest X-rays, whereas X-rays of 1.3% patients showed pulmonary edema. ECG findings showed sinus tachycardia in 32.8% of patients, sinus bradycardia in 18.4%, and tall T-waves in only 1.3%, whereas 47.4% had a normal sinus rhythm. Around 39.5% had metabolic acidosis, and 9.2% had metabolic alkalosis based on arterial blood gas analysis. A total of 28.9% of patients needed mechanical ventilation. It was found that 96% of the patients recovered and 2.6% succumbed to death. Also, a patient's average stay in the hospital was 4.83±2.4 days.

Conclusion

The research emphasizes the importance of prompt gastric lavage to mitigate the hazardous effects of amitraz. Despite the lack of an antidote, supportive treatment is necessary to address cardiac effects like bradycardia and hypotension, as well as central nervous system depression and respiratory failure. Even though amitraz intoxication has a benign prognosis, these cases are to be closely observed and monitored in intensive care units.

## Introduction

Amitraz, a compound belonging to the formamidine chemical family, functions as a 2-adrenergic agonist pharmacologically and as an acaricide and insecticide (1,5 di(2,4-dimethylphenyl)/3-methyl-1,3,5-triazapenta-1,4-diene) [1-2]. Intoxication can occur by oral, dermal, or inhalation routes [3]. Though its pharmacologic effects are derived from the inhibition of prostaglandin synthesis, 2-agonist adrenergic activation, and monoamine oxidase (MAO) inhibition, its clinical effects are typically linked to 2-agonist adrenergic activation [4]. Poisoning can cause a wide range of manifestations, including intestinal distension, pneumonia, hyperglycemia, polyuria, bradycardia, hypotension, hypothermia or fever, vomiting, and decreased gastrointestinal tract motility. It can also cause depression of the central nervous system (CNS), manifesting as mydriasis, miosis, or, in rare instances, drowsiness [5].

Xylene, an organic solvent in amitraz, can result in acute toxic symptoms, including episodes of neuro-irritability, nystagmus, ataxia, impaired motor coordination, stupor, and CNS depression [6-8]. Due to the underreporting of cases, there is currently very little literature available in our country. This is made worse by the fact that the toxidrome is misdiagnosed as organophosphate (OP) poisoning. Emergency medical personnel should be aware of this poisoning, its signs, and possible life-saving treatments as this poisoning usually recovers without any problems with early intervention [9]. Despite its prevalence, there is limited epidemiological data on amitraz poisoning in rural southern India. This study aims to fill this gap by analysing clinical presentations, outcomes, and management strategies in a tertiary care setting.

## Materials and methods

This was a retrospective study conducted between January 2014 and March 2024 at the Department of General Medicine of Shri B. M. Patil Medical College, Hospital and Research Centre (Vijayapura, Karnataka, India), which is a tertiary care hospital in southern India. The study was conducted with the help of the medical record section of the hospital. A total of 76 confirmed cases of amitraz poisoning that were admitted to our hospital between January 2014 and March 2024 were included. All patient-related information and investigations are kept up-to-date in the hospital's "NUMR" software and case files both. All the data required for this research was gathered from these sources. The Institutional Ethics Committee of Shri B. M. Patil Medical College, Hospital and Research Centre, BLDE (Deemed to be University), Vijayapura, issued approval BLDE (DU)/IEC/ 1078/ 2023-24.

Data collection

Patient data was extracted from hospital records, including symptoms, laboratory results, and outcomes, and was assessed based on the most common age group for exposure to poisoning, most common presentations, common signs, need for mechanical ventilation, duration of hospital stay, and also mortality rates.

Inclusion/exclusion criteria

All the poison detection center (PDC)-confirmed cases of amitraz poisoning, irrespective of age, admitted between January 2014 and March 2024 were included. Both male and female patients were included in the study. Poisoning cases involving other compounds along with amitraz were excluded.

Statistical analysis

The data obtained was entered in a Microsoft Excel sheet (Microsoft Corporation, Redmond, WA), and statistical analysis was performed using IBM SPSS Statistics, version 26.0 (IBM Corp., Armonk, NY), with a significance level set at p < 0.05. Results are presented as means ± standard deviations, frequency, percentage, median, and minimum and maximum values.

## Results

**Figure 1:**
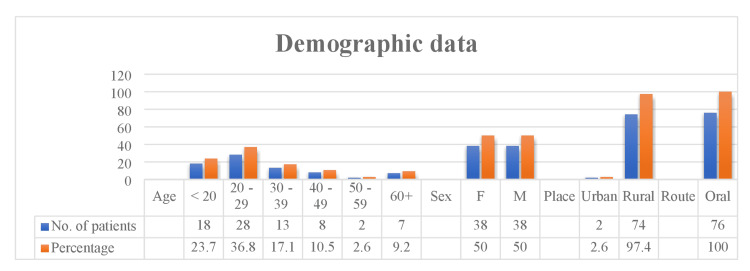
Basic demographic data of amitraz poisoning patients F, female; M, male

Figure 1 illustrates that the 20-29 year age group exhibited the highest frequency. Very few patients were above 60 years of age; there was no gender inequality seen our cases. All patients were from rural areas, and oral ingestion was the most typical mode of ingestion.

As shown in Table 1, the most common presenting symptom was vomiting, seen in 90.8% of patients, while miosis was the most frequent physical sign (73.7%). Notably, 28.9% of patients required mechanical ventilation, and the mortality rate was 2.6%.

**Table 1:** Clinical profile of amitraz poisoning DAMA, discharge against medical advice

		Frequency	Percentage
Time of presentation	AM	24	31.6
	PM	52	68.4
Symptoms			
Drowsiness	No	58	76.3
	Yes	18	23.7
Loss of consciousness	No	52	68.4
	Yes	24	31.6
Vomiting	No	7	9.2
	Yes	69	90.8
Signs			
Blood pressure	Hypotension	25	32.9
	Hypertension	8	10.5
	Normal	43	55.6
Temperature	Afebrile	64	84.2
	Hypothermia	12	15.8
Pupil	Mid-dilated	3	3.9
	Miosis	56	73.7
	Mydriasis	4	5.2
	Normal	13	17.1
Investigations			
Chest X-ray	Normal	69	90.8
	Pneumonitis	6	7.9
	Pulmonary edema	1	1.3
ECG	Sinus bradycardia	14	18.4
	Sinus tachycardia	25	32.8
	Sinus rhythm	36	47.4
	Tall t-waves	1	1.3
Arterial blood gas	Metabolic acidosis	30	39.5
	Respiratory acidosis	7	9.2
	Normal	39	51.3
Mechanical ventilation	No	54	71.1
	Yes	22	28.9
Outcome	DAMA	1	1.3
	Death	2	2.6
	Discharged	73	96.1

The mean age of patients in our study was 30.3 ± 14.27 years (Table 2). On laboratory investigations, blood glucose was found to be 148.61 ± 58.24 mg/dl. They were not known to be diabetic initially, and their blood sugar levels returned to normal on the subsequent follow-up without any treatment. Serum creatinine, sodium and potassium levels were within normal limits showing no renal involvement. Serum alanine aminotransferase (ALT), aspartate aminotransferase (AST) and alkaline phosphatase (ALP) levels were 28.72 ± 31.0 IU/l, 30.59 ± 24.59 IU/l and 88.01 ± 29.83 IU/l, respectively, and these findings suggested no significant hepatitis features seen. The overall length of hospital stay was 4.83 ± 2.46 days.

**Table 2:** Statistical values of important parameters of amitraz poisoning SpO_2_, oxygen saturation; RR, respiratory rate; GRBS, glucometer random blood sugar; Na^+^, serum sodium; K^+^, serum potassium; ALT, alanine aminotransferase; AST, aspartate aminotransferase; ALP, alkaline phosphatase

	Age (years)	Heart rate (beats/min)	SpO_2 _(%)	RR (cycles/min)	GRBS (mg/dl)	Creatinine (mg/dl)	Na^+^ (meq/dl)	K^+^ (meq/dl)	ALT (IU/l)	AST (IU/l)	ALP (IU/l)	Hospital stay (days)
Mean	30.30	82.83	95.00	17.09	148.61	0.73	142.88	4.39	28.72	30.59	88.01	4.83
Std. deviation	14.27	16.94	7.11	2.73	58.24	0.23	6.23	0.69	31.00	24.59	29.83	2.46
Minimum	15	41	45	12	63	0.4	133	2.5	10	11	48	1
Maximum	75	152	100	26	440	1.6	163	6.7	244	211	175	19

Management

Gastric lavage was performed on our patients when they initially arrived at the hospital, and then all the patients were admitted to the hospital's intensive care unit facility. Symptomatic treatment was given as there was no specific antidote for the poisoning. Hypotension responded to fluid therapy, and dopamine (5 μg/kg/min) infusion. Dopamine was administered to counteract bradycardia and hypotension, as it stimulates beta-adrenergic receptors to increase cardiac output. However, arterial blood gas analysis (ABG) revealed metabolic acidosis, a condition characterized by an excess of acid in the body fluids, observed in 39.5% of patients. Respiratory acidosis was seen in 9.2% of patients; these patients were intubated as per low oxygen saturation and mechanical ventilatory support was given in nearly one-third of patients (28.9%). Sinus tachycardia was seen in patients 32.2%, whereas 18.4% had sinus bradycardia and they responded to one to three doses of atropine IV (0.6 mg). Atropine accelerates the heart rate by blocking the cholinergic vagus nerve action, thereby increasing the rate of discharge by the sinus node and enhancing conduction through the atrioventricular node. The overall average hospital stay was 4.83 ± 2.46 days in our patients.

## Discussion

The minimum toxic dose for amitraz is considered to be 3.57 mg/kg in humans. The duration of action for oral poisoning is 30-120 min. Formamidines are toxic to humans and animals, and prior research has shown that these effects are reversible [1,10]. Intoxication can occur through the oral or dermal route or by inhalation [4]. There are not many documented cases in the literature of this pesticide intoxicating humans; what is known about it comes from isolated case reports or animal studies. Since amitraz functions as an α2-adrenergic agonist in the CNS and stimulates both α1- and α2-adrenergic receptors in the peripheral system, it has toxic effects. Additionally, prostaglandin E2 synthesis and the activity of the MAO enzyme are inhibited that leads to hypothermia [1]. Depending on the dosage, some of these effects might occur. While it rarely lasts longer than 48 hours, it has been demonstrated to have immediate toxic effects on both humans and animals [11,12].

The main clinical presentation is CNS depression with decreased spontaneous activity, miosis, bradycardia, hypotension, hypothermia, hyperglycemia, and respiratory depression that eventually leads to death. Complete recovery from all indications and symptoms happens in three to four days if treated promptly. Hyperreactivity to external stimuli may be a sign of amitraz toxicity at lower doses [1]. The results of this study align with previous findings on amitraz toxicity, but our data also revealed new insights regarding respiratory complications and the need for mechanical ventilation. This study analysed 76 cases of amitraz poisoning; among those, the rural population had the highest incidence rate (97.4%). It was most commonly observed in the 20-29 year age group (36.8% of patients); approximately 23.7% of patients were younger than 20 years old, whereas the incidence was lowest in patients over 60 years old (9.2%). Of the 76 cases, 50% were male patients, and the remaining 50% were female, suggesting no gender difference. As per the study by Demirel et al., the average age of the 45 amitraz poisoning cases was 30.41 ± 7.14 years, which is consistent with our study (30.3 ± 14.27 years) [4]. The only common mode of exposure observed in our patients was oral ingestion. It can also occur through accidental spraying or inhalation, also observed in a case series of 17 patients by Deepak et al. [13]. As per Table 1, the most frequent presentation was vomiting (90.8%) also observed in Yilmaz et al.'s study [6]. The high incidence of vomiting and miosis reinforces the need for prompt recognition of these signs in rural healthcare settings, where initial misdiagnosis as organophosphate poisoning is common. A total of 31.6% of patients presented with loss of consciousness, and drowsiness was seen in 23.7%.

As shown in Table 1, 32.9% of patients had hypotension and 10.5% of patients had hypertension. Our findings are consistent with those of Yilmaz and Yildizdas [6] and Aydin et al. [11] who found miosis and hypotension to be common signs of amitraz poisoning. Around 2.6% of patients had mydriasis as seen in a study by Rastogi et al. [14]. Only 15.8% of our patients had hypothermia, whereas 64.4% patients had hypothermia in the study by Demirel et al. [4]. A total of 7.9% of patients had pneumonitis, similar to that reported by Ulukaya et al. [13], and only one patient (1.3%) had pulmonary edema in our study. ABG analysis revealed metabolic acidosis in 39.5% of patients and respiratory acidosis in 9.2% of patients. However, our study was unique in highlighting a higher incidence of metabolic acidosis and the need for mechanical ventilation in nearly one-third (28.9%) of patients, whereas mechanical ventilation was not needed in any of the nine cases reported by Yilmaz and Yildizdas [6]. Sinus tachycardia was seen in 28.9% patients, whereas 18.4% had sinus bradycardia; a tall T wave was present in 1.3% of patients despite normal electrolytes. Overall, patients spent 4.83 ± 2.46 days on average in the hospital. Out of the 76 patients, approximately 96.1% recovered and were discharged successfully. The remaining 2.6% of patients passed away due to late presentation to the hospital, severe CNS depression that resulted in aspiration pneumonia, respiratory depression, and sudden cardiac death. Since there is no known counteragent for amitraz poisoning, medical care is mainly supportive and symptomatic [6,15]. Hemodynamic stabilization, airway maintenance [16], and steps to lessen the absorption of toxic materials must all be part of the strategy. Dopamine also has inotropic and chronotropic properties. Convincing evidence to support any inotrope as the recommended first-line treatment is lacking, as there are very few case reports on the use of inotropes in amitraz poisoning. Dopamine, as administered in our patient, can be used at doses of 5-10 mg/kg/min as an inotrope to counteract the bradycardia and hypotension brought on by amitraz [17].

Limitations

This study also had certain limitations. This was a single-centre retrospective study with a limited sample size; further multi-centric studies with larger sample sizes and prospective studies will help and improve the early diagnosis and management of amitraz poisoning in a better way.

## Conclusions

In documented cases of amitraz poisoning in humans, recovery is typically observed within 12-48 hours despite a potentially fatal clinical picture involving CNS and cardiac depression. Since there is not a specific antidote or set of management guidelines, doctors rely on prior case reports and review articles for assistance in managing patients. Treatment for amitraz poisoning consists of supportive and symptomatic measures, such as monitoring of the respiratory, cardiovascular, and neurological systems. While amitraz poisoning generally has a good prognosis, this study highlights the need for early intervention, especially in cases presenting with metabolic acidosis and respiratory depression. Future studies should focus on optimizing the management protocols and exploring possible antidotes.

## References

[1] Agin H, Calkavur S, Uzun H, Bak M (2004). Amitraz poisoning: clinical and laboratory findings. Indian Pediatr.

[2] Jorens PG, Zandijk E, Belmans L, Schepens PJ, Bossaert LL (1997). An unusual poisoning with the unusual pesticide amitraz. Hum Exp Toxicol.

[3] Ertekin V, Alp H, Selimoğlu MA, Karacan M (2002). Amitraz poisoning in children: retrospective analysis of 21 cases. J Int Med Res.

[4] Demirel Y, Yilmaz A, Gursoy S, Kaygusuz K, Mimaroglu C (2006). Acute amitraz intoxication: retrospective analysis of 45 cases. Hum Exp Toxicol.

[5] Dreisbach RH (1977). Handbook of Poisoning. Diagnosis and Treatment. California: Lange Medical Publishing.

[6] Yilmaz HL, Yildizdas DR (2003). Amitraz poisoning, an emerging problem: epidemiology, clinical features, management, and preventive strategies. Arch Dis Child.

[7] Avsarogullari L, Ikizceli I, Sungur M, Sözüer E, Akdur O, Yücei M (2006). Acute amitraz poisoning in adults: clinical features, laboratory findings, and management. Clin Toxicol (Phila).

[8] Jones RD (1990). Xylene/amitraz: a pharmacologic review and profile. Vet Hum Toxicol.

[9] Gursoy S, Kunt N, Kaygusuz K, Kafali H (2005). Intravenous amitraz poisoning. Clin Toxicol (Phila).

[10] Ulukaya S, Demirag K, Moral AR (2001). Acute amitraz intoxication in human. Intensive Care Med.

[11] Aydin K, Per H, Kurtoglu S, Poyrazoglu MH, Narin N, Aslan D (2002). Amitraz poisoning in children. Eur J Pediatr.

[12] Prajapati T, Patel N, Zamani N, Mehrpour O (2012). Amitraz poisoning: a case study. Iran J Pharmacol Ther.

[13] Deepak K, Rao N, Devi R, Reddy M, Bhaskar J (2021). Case series: amitraz poisoning: clinical profile and management: is stomach wash an antidote??. Int J Adv Res.

[14] Rastogi A, Joshi A (2018). The clinical profile and complications of amitraz poison, a near-fatal poisoning. Int J Recent Sci Res.

[15] Veale DJ, Wium CA, Muller GJ (2011). Amitraz poisoning in South Africa: a two year survey (2008-2009). Clin Toxicol (Phila).

[16] Elinav E, Shapira Y, Ofran Y, Hassin T, Ben-Dov IZ (2005). Near-fatal amitraz intoxication: the overlooked pesticide. Basic Clin Pharmacol Toxicol.

[17] Herath HM, Pahalagamage SP, Yogendranathan N, Wijayabandara MD, Kulatunga A (2017). Amitraz poisoning: a case report of an unusual pesticide poisoning in Sri Lanka and literature review. BMC Pharmacol Toxicol.

